# Resistant Gram-negative infections in the outpatient setting in Latin America

**DOI:** 10.1017/S095026881300191X

**Published:** 2013-08-07

**Authors:** M. J. C. SALLES, J. ZURITA, C. MEJÍA, M. V. VILLEGAS

**Affiliations:** 1 Internal Medicine Department, Santa Casa de São Paulo School of Medicine, São Paulo, Brazil; 2 Hospital Vozandes, Facultad de Medicina, Pontificia Universidad Católica del Ecuador, Quito, Ecuador; 3 Infectious Diseases Unit, Hospital Roosevelt, Guatemala City, Guatemala; 4 Bacterial Resistance Group, International Center for Medical Research and Training (CIDEIM), Cali, Colombia

**Keywords:** Drug resistance, Gram-negative, intra-abdominal infection, Latin America, outpatient, urinary tract infection

## Abstract

Latin America has a high rate of community-associated infections caused by multidrug-resistant Enterobacteriaceae relative to other world regions. A review of the literature over the last 10 years indicates that urinary tract infections (UTIs) by *Escherichia coli*, and intra-abdominal infections (IAIs) by *E. coli* and *Klebsiella pneumoniae*, were characterized by high rates of resistance to trimethoprim/sulfamethoxazole, quinolones, and second-generation cephalosporins, and by low levels of resistance to aminoglycosides, nitrofurantoin, and fosfomycin. In addition, preliminary data indicate an increase in IAIs by Enterobacteriaceae producing extended-spectrum *β*-lactamases, with reduced susceptibilities to third- and fourth-generation cephalosporins. Primary-care physicians in Latin America should recognize the public health threat associated with UTIs and IAIs by resistant Gram-negative bacteria. As the number of therapeutic options become limited, we recommend that antimicrobial prescribing be guided by infection severity, established patient risk factors for multidrug-resistant infections, acquaintance with local antimicrobial susceptibility data, and culture collection.

## INTRODUCTION

Antibacterial resistance in clinically important Gram-negative pathogens is transmitted easily among individuals in various community settings via water, sanitation, hygiene, and food pathways [1–6]. In both urban and rural settings, there are many means by which resistant pathogens are disseminated, including human migration, overcrowding, chemical pollution, waste water, and untreated groundwater [3, 5–9]. An important mode of transmission of resistant Gram-negative bacteria includes animal vectors, as observational data have clearly shown a similarity in the resistance genes and enterotoxins of *Escherichia coli* isolated from companion animals and horses, and extended-spectrum *β*-lactamase (ESBL)-producing isolates described in humans [10]. Faecal carriage of antibiotic-resistant pathogens in humans and animals predisposes to further contamination of food and water supplies to complete a transmission cycle [4, 11–16].

Latin America fulfils all of the above-mentioned criteria required to drive the spread of antibacterial drug resistance. As in other world regions, high antibacterial use and misuse (e.g. inappropriate drug selection, suboptimal dosing, poor patient adherence) may also drive bacterial resistance in Latin America [7, 17]. Consequently, for many pathogens, rates of antimicrobial resistance in Latin America appear to be high relative to other regions of the world [8, 18].

The unique set of medical, societal, and ecological circumstances in Latin America has underpinned a dynamic epidemiology of Gram-negative infections in the outpatient setting over the last 15 years. In particular, community-associated infections caused by multidrug-resistant Enterobacteriaceae and other Gram-negative bacteria are an important public health concern [19–25]. Foremost of the pathogens causing concern are strains of *E. coli*-producing ESBLs, which are an important cause of urinary tract infection (UTI) and intra-abdominal infection (IAI) sometimes associated with bacteraemia [19–21, 23, 26, 27]. Because ESBL-producing bacteria now cause many infections in the community setting, the medical community is increasingly reliant on multilevel microbial surveillance to inform treatment decisions, identify major problems, and guide adequate control measures [19–22, 26, 28].

This narrative review stems from the 10th Meeting of the Latin America Working Group on Bacterial Resistance in São Paulo, Brazil, 20–21 May 2012, at which the Working Group members reflected on the increasing recognition of the clinical importance of multidrug-resistant Gram-negative infections in the community setting in Latin America. Using primary data from published studies and policy documents, the aim of this review is to report on the epidemiology of Gram-negative bacteria isolated in Latin America over the last 10 years, describe the importance of detection in outpatients, and consider the implications for prescribing decisions.

## METHODS

In order to review the published clinical data relating to UTIs and IAIs due to Gram-negative pathogens in the community setting of Latin America, a systematic search of the biomedical literature was conducted. The title/abstract fields of Pubmed were searched, limited by the dates 1 January 2005 to 12 November 2012, for articles using the following terms and Boolean logic: (‘Latin America’ or ‘South America’ or ‘Central America’ or Mexico or Guatemala or Honduras or Nicaragua or ‘Costa Rica’ or Cuba or ‘Dominican Republic’ or Panama or Colombia or Venezuela or Guyana or Suriname or ‘French Guiana’ or Brazil or Ecuador or Peru or Bolivia or Paraguay or Uruguay or Chile or Argentina) and (‘Gram-negative infection’ or ‘Gram-negative pathogen’ or ‘Gram-negative bacilli’ or ‘*Escherichia coli*’ or ‘*Klebsiella pneumoniae*’ or ‘*Proteus mirabilis*’ or *Citrobacter* or *Serratia* or ‘urinary tract infection’ or ‘intra-abdominal infection’). In addition, we searched the Scientific Electronic Library Online (SciELO), which publishes health science data specifically from Latin America. Searches on SciELO were limited to policy statements. A small number of additional references were identified from the reference lists of published articles.

The titles and abstracts of all references obtained in the search were screened by the authors. We included all original research articles that reported information on the: (1) susceptibilities of Gram-negative pathogens causing UTIs and IAIs in Latin American primary care; (2) proportion of Gram-negative clinical isolates harbouring ESBL genes or expressing ESBLs; and (3) presence of circulating ESBLs and first reports of novel ESBLs. Only studies reporting data for >40 isolates were selected for assessing drug resistance.

## EPIDEMIOLOGY

### Overview

**Fig. 1.**
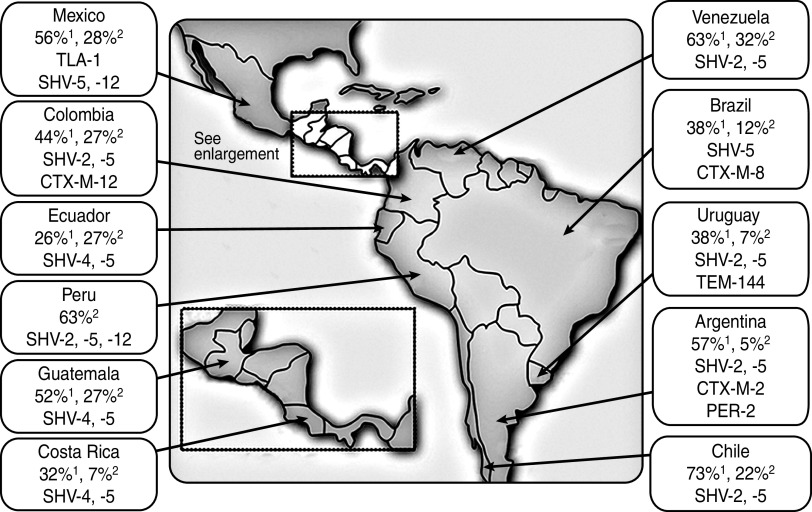
Prevalence (and type) of extended spectrum *β*-lactamases harboured by *K. pneumoniae*^1^ and *E. coli*^2^ in Latin American clinical isolates during the late 1990s [32].

ESBLs are found predominantly in *Klebsiella* spp. and *E. coli*, but have also been described in other genera of Enterobacteriaceae including species of *Citrobacter, Serratia, Proteus, Salmonella,* and *Enterobacter* [29, 30]. Until the turn of the century, *Klebsiella pneumoniae* harbouring Temoneira (TEM)-type and sulfhydryl variable (SHV)-type ESBLs were prevalent in the nosocomial setting only, and cefotaxime-resistant (CTX-M) *β*-lactamase-producing organisms were rarely isolated (Fig. 1) [30–32]. However, in the first years of this century, Latin America became the first continent where CTX-M variants began to displace TEM and SHV variants as the most common type of ESBL, mainly in *E. coli* [31]. CTX-M-type ESBLs share only 40% homology with TEM or SHV enzymes and are considered unrelated [30].

The CTX-M-type ESBLs have now reached endemic proportions in South America aided by the location of *bla*_CTX-M_ genes on plasmids and transposons, which engenders dissemination of CTX-M-producing strains in different Enterobacteriaceae [30, 31, 33]. The spread of mobile genetic elements, mainly conjugative plasmids belonging to classic incompatibility groups, and the dispersion of specific clones have been responsible for the increase in ESBL-producing isolates and for the spread of many specific ESBLs, including CTX-M. ESBLs are often associated with co-resistance to fluoroquinolones, aminoglycosides, and trimethoprim/sulfamethoxazole, which may also have contributed to the current epidemiological ESBL scenario [33].

Also over the same time period, *E. coli* replaced *Klebsiella* spp. as the predominant species of ESBL-producing Enterobacteriaceae in the community, largely because of the ease of CTX-M mobilization [31]. Perhaps the high prevalence of community-acquired infection by *E. coli-*harbouring ESBLs was to be expected, given that these strains have been isolated from numerous sources such as domestic animals, food products, well water, sewage, and stool samples from healthy individuals [6, 34]. Importantly, there is evidence that community-associated *E. coli* ESBLs have infiltrated hospital settings [31, 34].

The most common community-acquired infection by Gram-negative bacteria is UTI, but clinicians are confronted with many different infection types including pneumonia and IAI [19, 20, 35]. *E. coli, K. pneumoniae*, and *Proteus mirabilis* are the most common organisms causing UTIs in ambulatory patients in Latin America [19–21, 35]. The aetiology of community-associated IAIs depends on the distribution of microflora at the anatomical site, although infections by enterobacteria (primarily *E. coli* and *K. pneumoniae*) tend to predominate [36].

### UTI

**Fig. 2.**
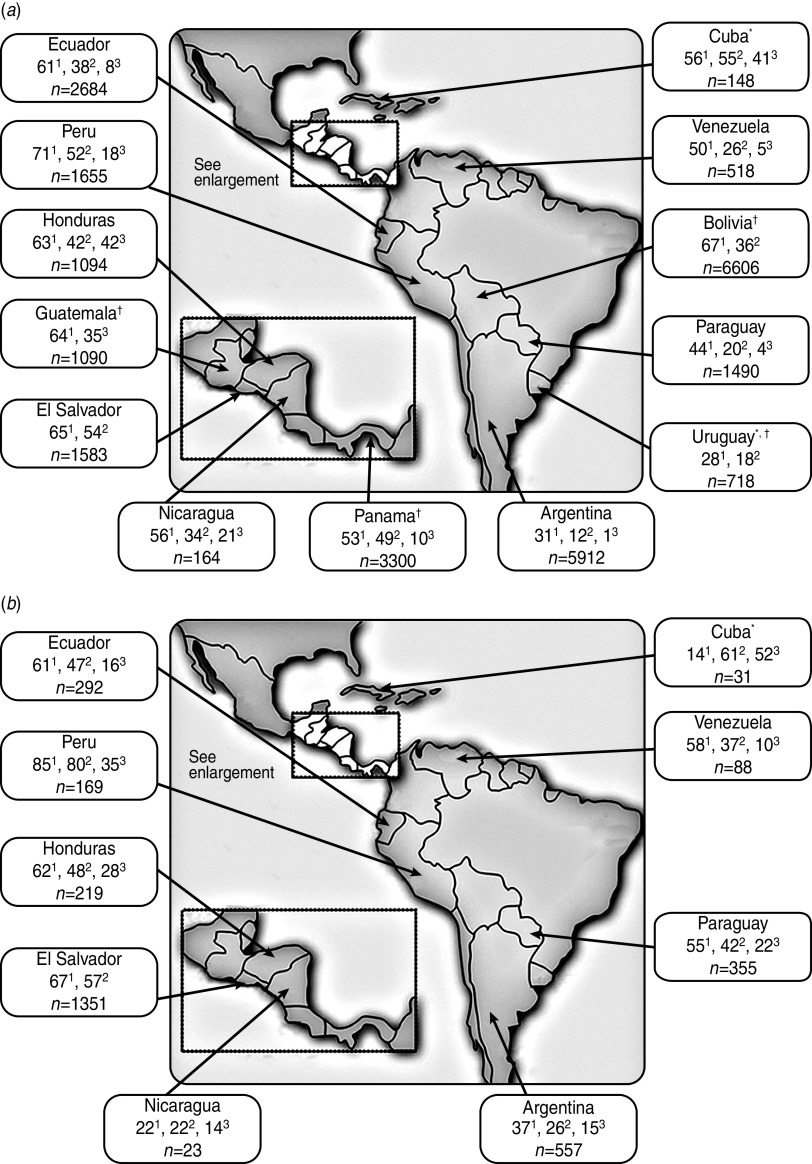
Percentage (no. of isolates) of urinary tract *E. coli* isolates collected from (*a*) women and (*b*) men in Latin America (2010 PAHO report) that were resistant to trimethoprim/sulfamethoxazole^1^, ciprofloxacin^2^, and the second-generation cephalosporin cefuroxime^3^ [39]. * Data from the 2009 PAHO report [38] is given for this country because 2010 data were not reported; † all adults (women and men).

The current rate of clinical failure associated with community-source uropathogens is unacceptably high, coincident with high levels of resistance to commonly used antimicrobials (Table 1, Fig. 2) [19, 25, 37–41]. Overall, data from four network studies [19, 25, 40, 41] as well as data from the Pan-American Health Organization (PAHO) surveillance system for 2009 and 2010 [38, 39], indicated that continent-wide resistance of *E. coli* to trimethoprim/sulfamethoxazole was high, resistance to quinolones was variable, and resistance to second-generation cephalosporins and gentamicin was routinely greater than 20%. In contrast, rates of *E. coli* resistance to nitrofurantoin and fosfomycin were generally low.

**Table 1.** Percentage of drug-resistant community-acquired urinary tract E. coli isolates collected during five surveillance network studies

Antimicrobial	Country (network), year (no. of isolates)				
	Guatemala [40]	Ecuador (REDNARBEC) [25]		Chilean network [41]	Argentina, Brazil, Chile, Mexico, Venezuela (SENTRY) [19]
	2005–2010 (*n* = 1184)	1999 (*n* = 1907)	2007 (*n* = 6447)	2009 (*n* = 36 949)	2003 (*n* = 403)
Ampicillin		57	72	53·6 n.s.	53·6
Ampicillin/sulbactam	29·3	19	27		23·3
Amoxicillin/clavulanate					1·2
Piperacillin/tazobactam	2·9			6·4 n.s.	0·2
C1G		25*, 8†	29*, 9†	31·8* n.s.	
C2G		3‡	9‡		2·2‡
C3G	2·5§, 10·8||	1¶	3¶	5·6||	1·5§, 1·2¶
C4G	8·3#				1·0#
Cefoxitin					1·7
Gentamicin	21·9	3	18	6·9 n.s.	8·4
Amikacin	1·4	0·5	0·9	7·5 n.s.	0
Ciprofloxacin	46·3	18	41	19·6 n.s.	21·6
Trimethoprim/sulfamethoxazole	58·6	50	57	29·6 n.s.	40·4
Nitrofurantoin	2·5	4	7	4 n.s.	6·9
Ertapenem				0 n.s.	
Imipenem	0			0 n.s.	0
Meropenem	0			0 n.s.	
Fosfomycin		1	2		

REDNARBEC, Red Nacional de Resistencia Bacteriana de Ecuador; C1G, First-generation cephalosporins; C2G, second-generation cephalosporins; C3G, third-generation cephalosporins; C4G, fourth-generation cephalosporins; n.s., non-susceptible.

* Cefalotin; † cefazolin; ‡ cefuroxime; § ceftazidime; || cefotaxime; ¶ ceftriaxone; # cefepime.

Rates of ESBL production by community-source isolates have been reported only infrequently in the literature. In the SENTRY 2003 study, which collected data from Argentina, Brazil, Chile, Mexico and Venezuela, rates of ESBL production were 1·7%, 16·3%, and 5·1% in urinary isolates of *E. coli, Klebsiella* spp., and *P. mirabilis*, respectively [19]. The rate of ESBL production in *E. coli* isolates was 16% in outpatients in Guatemala during the period 2005–2010 [40].

#### Central America

The PAHO 2010 data for isolates of community origin reported high resistance rates of *E. coli* to trimethoprim/sulfamethoxazole and ciprofloxacin throughout Central America (Fig. 2), as well as low resistance rates of *E. coli* to nitrofurantoin (⩽17%). Resistance of *E. coli* to cefuroxime ranged from 10% (in Panama) to 42% (females in Honduras); resistance to gentamicin ranged from 4% (in Guatemala) to 28% (males in Honduras) [39]. In the 4 years since PAHO 2006 surveillance data were collected, PAHO 2010 surveillance data indicated that resistance had edged higher to most antimicrobial drug classes in those countries with available data (i.e. El Salvador, Honduras, Nicaragua).

Resistance rates of *E. coli* isolates collected in a surveillance study in Guatemala in 2005–2010 are shown in Table 1. In Mexican data from the SENTRY 2003 study, antimicrobial susceptibility rates of urinary *E. coli* isolates were as follows: ampicillin, 22%; nalidixic acid, 25%; levofloxacin, 28%; gatifloxacin, 31%; trimethoprim/sulfamethoxazole, 39%; amoxicillin/clavulanate, 56%; cefuroxime, 64%; and nitrofurantoin, 81% [19]. Although the nitrofurantoin susceptibility data in SENTRY 2003 data for Mexico were encouraging, increased utilization of this antimicrobial agent as a recommended first-line agent appeared to induce resistance in a study of 304 patients with suspicion of UTI at the university hospital and primary health centres of León, Nicaragua [20]. In the 5 years after the introduction of the therapeutic guidelines, resistance of *E. coli* against nitrofurantoin increased from 0% to 7% [20]. In this study [20], high resistance rates of *E. coli* were also observed in 2008 with ampicillin (61%), cefalotin (46%), trimethoprim/sulfamethoxazole (39%), ciprofloxacin (32%), gentamicin (25%), and ceftriaxone (20%). Thirteen (30%) of 44 *E. coli* strains were suspected of producing ESBLs, with resistance rates in ESBL-producing *E. coli* significantly higher to ampicillin (85% *vs*. 52%), amoxicillin/clavulanate (46% *vs*. 6%), cefalotin (85% *vs*. 29%), ceftriaxone (69% *vs*. 0%), and ciprofloxacin (62% *vs*. 19%) compared to pathogens that did not produce ESBLs [20].

#### South America

The PAHO 2010 data for isolates of community origin revealed that resistance of *E. coli* to trimethoprim/sulfamethoxazole ranged from 31% to 85% across South American countries, and resistance to ciprofloxacin ranged from 12% to 80%, with the lowest and highest resistance rates for both antimicrobial agents occurring in Argentina and Peru (Fig. 2) [39]. *E. coli* resistance to nitrofurantoin was low (i.e. <20%) except in Argentinian males (31%). In contrast, resistance to gentamicin was erratic, ranging from 4% in Uruguay to 29% in Peruvian males [39].

Eight-year temporal data reported in the Red Nacional de Resistencia Bacteriana de Ecuador (REDNARBEC) study are shown in Table 1. Of the South American countries that participated in SENTRY 2003 (Argentina, Brazil, Chile, Venezuela; Table 1), susceptibility rates were generally comparable across countries [19]. The greatest difference was for the fluoroquinolones, with susceptibility to levofloxacin ranging from 72% in Venezuela to 91% in Brazil [19].

Results of a large Brazilian study conducted in Curitiba found that few suitable empirical treatment options for community-source UTIs were available for women aged >60 years and males of any age [37]. Of 9798 consecutive, non-duplicate, community-source urine isolates from ambulatory patients aged >13 years during 2009, *E. coli* (66%) was by far the most prevalent Gram-negative pathogen followed by *Klebsiella* spp. (6%), *P. mirabilis* (4%), and *Enterobacter* spp. (3%) [37]. Susceptibility of *E. coli* varied widely by drug class, being very low for ampicillin and trimethoprim/sulfamethoxazole (56% and 66%, respectively), suboptimal for fluoroquinolones (82%), and acceptable for gentamicin, ceftriaxone/cefotaxime and nitrofurantoin (93%, 97% and 96%, respectively) [37]. Importantly, the susceptibility rates of *E. coli* urinary isolates were 3–4% higher for fluoroquinolones and gentamicin, and at least 30% higher for nitrofurantoin and extended-spectrum cephalosporins, compared to susceptibility rates for other pathogens causing community-source urinary infections [37].

### IAI

The Study for Monitoring Antimicrobial Resistance Trends (SMART) monitors the activity of key antimicrobial drug classes against Gram-negative bacteria isolated from IAIs to ensure that the current susceptibility patterns of these organisms are well understood and reported effectively [36]. The number of participating sites in Latin America increased from six in 2002 to 13 in 2007, with 740 isolates sent for analysis on average each year [36]. In 2008, there were 23 centres in ten Latin American countries (Argentina, Brazil, Chile, Colombia, Dominican Republic, Guatemala, Mexico, Panama, Peru, Venezuela) that participated in SMART, with each centre collecting up to 100 consecutive non-duplicate clinical isolates from patients with IAI [42]. Of 1003 Gram-negative bacilli isolates collected from IAIs, *E. coli* (50%), *K. pneumoniae* (15%), and *Enterobacter cloacae* (7%) were the most commonly isolated pathogens (4% of isolates were *P. mirabilis*) [42]. More than one quarter of *E. coli* (27%) and more than one third of *K. pneumoniae* (38%) isolates were ESBL-positive. The prevalence of ESBL-producing *E. coli* and *Klebsiella* spp. associated with community-acquired infections in particular was 29% [42].

**Fig. 3.**
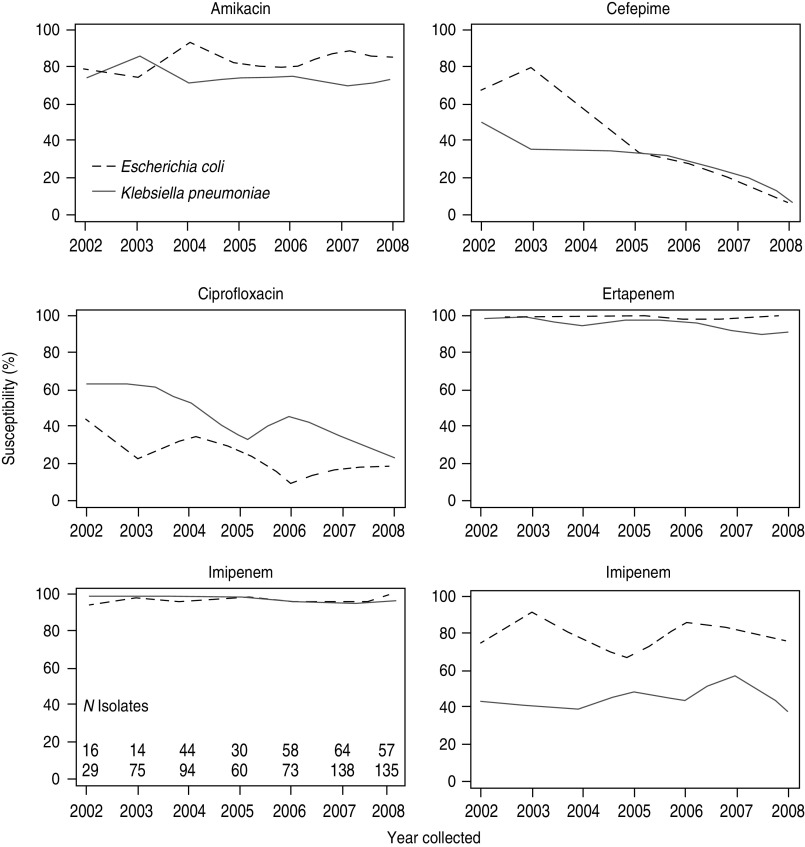
Antimicrobial susceptibilities of ESBL-producing *E. coli* and *K. pneumoniae* intra-abdominal isolates in Latin America (2002–2008). Susceptibilities are based on *in vitro* minimum inhibitory concentration data. (Reprinted from Villegas *et al*. [42], copyright © 2011, with permission from Elsevier.)

In the SMART study, susceptibilities of ESBL-producing strains to antibacterial agents commonly used in the community such as third-generation cephalosporins, fluoroquinolones, and ampicillin/sulbactam were low (Table 2) [42]. Six-year temporal data from SMART indicate that the percentages of susceptible ESBL-positive *E. coli* and *K. pneumoniae* isolates to ciprofloxacin and cefepime are declining, whereas susceptibilities to amikacin are steady (Fig. 3) [42].

**Table 2.** Antimicrobial susceptibilities of the most commonly isolated pathogens (>50 isolates) recovered from intra-abdominal infections of Latin American patients participating in SMART, 2008 [42]

	Amikacin	Ampicillin/sulbactam	Cefepime	Cefotaxime	Cefoxitin	Ceftazidime	Ceftriaxone	Cipro-floxacin	Ertapenem	Imipenem	Levofloxacin	Piperacillin/tazobactam
*Enterobacter cloacae*	97·1	17·4	84·1	55·1	2·9	59·4	52·2	87	98·6	100	94·2	68·1
*Escherichia coli*, ESBL-positive	85·2	2·2	7·4	1·5	76·3	28·1	1·5	19·3	99·3	100	20	77·8
*Escherichia coli*, non-ESBL	98·1	48·5	100	96·5	91·3	95·9	95·7	74·8	99·7	99·5	75·9	91·1
*Klebsiella pneumoniae*, ESBL-positive	71·9	0	8·8	7	61·4	14	3·5	22·8	91·2	98·2	35·1	40·4
*Klebsiella pneumoniae*, non-ESBL	92·6	59·6	96·8	96·8	87·2	96·8	95·7	79·8	96·8	98·9	83	84

ESBL, Extended-spectrum *β*-lactamase.

Reprinted from Villegas *et al*. [42], copyright © 2011, with permission from Elsevier.

**Fig. 4.**
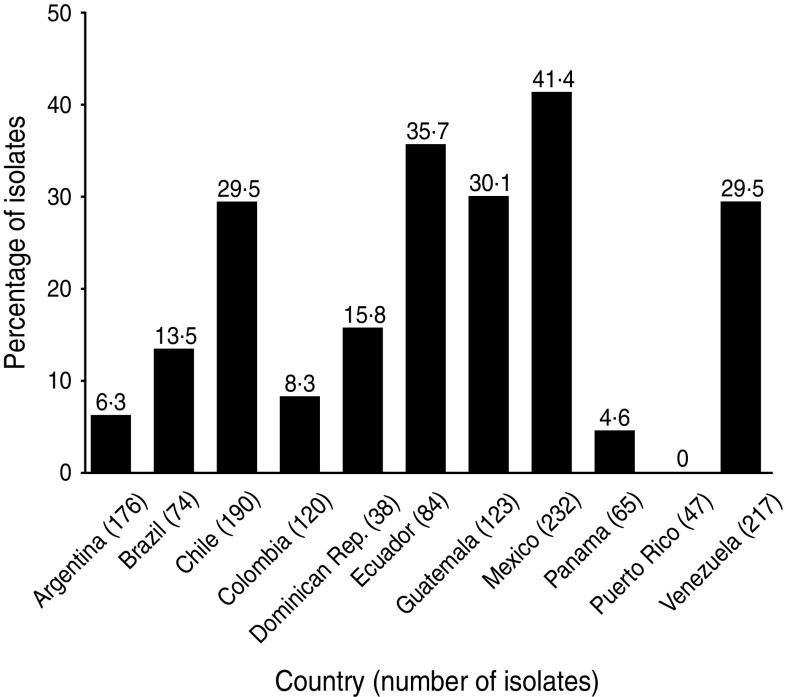
Proportion of *E. coli* isolates from intra-abdominal infections in Latin America that were extended-spectrum *β*-lactamase positive (SMART, 2008–2009) [43].

The phenotypes of *E. coli* isolated from 19 SMART investigator sites in 11 Latin American countries during 2008–2009 were determined [43]. Of the 1366 isolates, 323 (24%) produced ESBLs, which is an increase from 12% in 2004 and 22% in 2005–2007. The proportion of isolates that were ESBL-producing varied widely in Latin American countries (Fig. 4) [43]. ESBL production had a major deleterious effect on activity against ampicillin/sulbactam, cephalosporins, and fluoroquinolones, as well as on the activity of amikacin, perhaps indicating a co-resistance phenomenon (Table 3) [43]. It has been postulated that fluoroquinolone resistance is a harbinger of broader antimicrobial resistance, including ESBL selection [44].

**Table 3.** Antimicrobial susceptibilities of 323 ESBL-positive intra-abdominal E. coli isolates tested in SMART 2008–2009 based on Clinical and Laboratory Standards Institute breakpoints [43]

Antimicrobial	MIC_90_ (*μ*g/ml)	Susceptible (%)	Intermediate susceptible (%)	Resistant (%)
Ampicillin/sulbactam	>16 (>16)	6·8 (42·8)	23·5 (23·1)	69·7 (34·1)
Piperacillin/tazobactam	64 (16)	80·5 (91·7)	12·7 (3·2)	6·8 (5·1)
Cefepime	>32 (⩽0·5)	11·8 (100)	2·2 (0)	86·0 (0)
Cefotaxime	>128 (⩽0·5)	0·9 (94·3)	0·9 (1·0)	98·2 (4·7)
Cefoxitin	16 (8)	79·6 (92·2)	14·5 (1·7)	5·9 (6·1)
Ceftazidime	>128 (⩽0·5)	21·4 (95·1)	4·0 (1·1)	74·6 (3·8)
Ceftriaxone	>32 (⩽1)	0·9 (94·1)	0·6 (0·7)	98·5 (5·2)
Amikacin	32 (8)	88·8 (98·4)	5·6 (1·2)	5·6 (0·5)
Ciprofloxacin	>2 (>2)	16·4 (69·2)	0·0 (0·6)	83·6 (30·2)
Levofloxacin	>4 (>4)	17·3 (70·4)	1·6 (2·1)	81·1 (27·5)
Ertapenem	0·25 (⩽0·03)	95·1 (99·1)	4·0 (0·8)	0·9 (0·1)
Imipenem	0·25 (0·12)	99·4 (99·3)	0·3 (0·4)	0·3 (0·3)

ESBL, Extended-spectrum *β*-lactamase.

Values in parentheses are the corresponding data pertaining to the 1043 ESBL-negative isolates tested in SMART 2008–2009.

## RISK FACTORS

Early recognition of patients at a heightened risk for infection with multidrug-resistant bacteria is necessary to provide appropriate empirical treatment and institute measures that control or limit disease transmission. This observation is especially true in cases of IAI since ampicillin/sulbactam, third-generation cephalosporins, and fluoroquinolones have a limited role as first-line treatments for IAIs. Risk factors for infection with ESBL-producing Enterobacteriaceae have been identified in several studies of hospitalized patients [45–49]. Risk factors identified in multivariate analyses in these studies include previous antibiotic use (specifically, use of quinolones [47], cephalosporins [45], oxyimino-cephalosporins [46], piperacillin/tazobactam [45], or *β*-lactams with an oxyimino group [48]), recurrent infections [47], haemodialysis [49], urinary catheterization [46], artificial nutrition [47], and residence in a nursing home [49].

The relevance of risk factors in the hospital setting to the community setting is unclear. According to Pitout & Laupland [26], repeated episodes of UTI and underlying renal pathology, previous use of antibiotics including cephalosporins and quinolones, previous hospitalization, nursing home residence, older age, presence of diabetes mellitus, and underlying liver pathology are risk factors for community-onset infections caused by ESBL-producing bacteria [26]. A meta-analysis of epidemiological studies of infection caused by ESBL-producing Enterobacteriaceae in non-hospitalized patients from six centres in Europe, Asia, and North America revealed that recent use of any antibiotic, residence in a long-term care facility, recent hospitalization, age ⩾65 years, and male sex were statistically significant risk factors for infection by an ESBL-producing organism [34]. However, it can be argued that emergence of ESBL-producing bacteria, primarily CTX-M-type *β*-lactamases, confounds infection control strategies based on traditional risk factors, since one third of ESBL-producing isolates in the meta-analysis were obtained from patients with no recent healthcare contact [34]. Multivariate analysis of a nested case-control study of 787 consecutive patients presenting with febrile UTI in 2004–2009 in The Netherlands revealed recent hospitalization, presence of a urinary catheter, and fluoroquinolone use in the past 6 months to be independent risk factors for infection due to fluoroquinolone-resistant *E. coli* [44]. In summary, risk factors for infection by ESBL-producing bacteria in the community setting are similar to those in the hospital setting and are fairly consistent across studies.

## SURVEILLANCE AND DETECTION

With the advent of widespread dispersion of ESBL-producing Enterobacteriaceae in communities of Latin America, empirical prescribing of antimicrobial agents according to general principles can no longer ensure effective treatment. Rather, local susceptibility patterns attained within each institution and maintenance of longitudinal surveillance programmes are needed to inform treatment decisions. Surveillance also aids patient diagnosis and facilitates infection control strategies. Despite an investment to survey bacterial resistance in Latin America through PAHO, SENTRY, SMART and other programmes, further efforts are required to more fully integrate these resources in a practical way so that real-time data regarding antimicrobial susceptibility and genotypic patterns are available to healthcare professionals [28].

Aside from these overarching logistical issues, there are also major limitations in our capability to detect ESBL-producing Enterobacteriaceae. These strains confound traditional surveillance strategies because ESBL production cannot be inferred from the antimicrobial resistance profile alone. Hence, the emergence, maintenance, and dissemination of ESBLs must be characterized and closely monitored by implementation of integrated phenotypic and genotypic testing strategies. To give some scale to the magnitude of the task ahead, the number of CTX-M variants alone numbered 138 as of March 2013 [50].

### Phenotypic methods

Most clinical microbiology laboratories in Latin America conduct susceptibility testing as it is easy, automated, inexpensive, and accessible; however, phenotypic methods cannot provide information on the type of ESBL produced. The value of non-molecular phenotypic methods is premised on the fact that most ESBLs hydrolyse third-generation cephalosporins and are inhibited by clavulanate [26]. Numerous methods have been developed to detect or confirm ESBL production by Enterobacteriaceae. Automated systems screen isolates and confirm ESBL production based on minimum inhibitory concentrations of cefotaxime and ceftazidime with and without clavulanic acid.

However, the most appropriate choice of extended-spectrum cephalosporins and type of confirmatory test that provides the greatest assay sensitivity have yet to be determined. For instance, in early testing, the E-test ESBL screen test with ceftazidime or cefepime as substrate, and use of Oxoid combination discs with cefotaxime and ceftazidime, were useful for demonstrating the presence of Enterobacteriaceae potentially producing ESBLs but were also associated with clinically significant false-susceptible and false-resistant results [51, 52]. Current guidance from the Clinical and Laboratory Standards Institute (CLSI) describes laboratory detection of ESBL produced by *E. coli, P. mirabilis*, and *Klebsiella* spp., but not for species with inducible AmpC *β*-lactamases (such as *Enterobacter* spp.) [53]. Specifically, for ESBL detection in Enterobacteriaceae, CLSI recommends initial screening with 8 *μ*g/ml of cefpodoxime; 1 *μ*g/ml each of cefotaxime, ceftazidime, ceftriaxone, or aztreonam; followed by confirmatory tests (including the E-test ESBL strips) with both cefotaxime and ceftazidime in combination with clavulanate at a concentration of 4 *μ*g/ml [54]. As of 2007, high sensitivities of up to 94% and specificity of 98% for detecting ESBLs in *E. coli, Klebsiella* spp, and *Proteus* spp. were expected if these techniques were adhered to [55].

Recent emergence of plasmidic AmpC *β*-lactamases harboured by *E. coli* and *K. pneumoniae* may result in changes to CLSI recommendations. The phenotypic detection of ESBLs in bacteria other than *E. coli, Klebsiella* spp., and *Proteus* spp. will remain problematical because of the possible association of resistance mechanisms, presence of more than one *β*-lactamase [especially carbapenemases of the *K. pneumoniae* carbapenemase (KPC) type], and reduced permeability.

### Genotypic methods

Genotypic methods use molecular biology techniques to detect the gene responsible for ESBL production, with the aim of distinguishing between resistance genes, genetic elements, and strains. The primary technology used is polymerase chain reaction (PCR) amplification of *bla*_TEM_, *bla*_SHV_, and bla_CTX-M_ genes with oligonucleotide primers followed by sequencing to discriminate between non-ESBL parent enzymes and their ESBL variants [26]. ESBLs have also been characterized by PCR restriction fragment-length polymorphism and single-strand conformational polymorphism, and by restriction site insertion PCR, real-time PCR, and ligase chain reaction [26]. A limitation of PCR is that it detects ESBL genes but does not inform on ESBL production. In addition, few clinical microbiology laboratories in Latin America are equipped to perform recombinant DNA techniques, which are complicated, time-consuming, and expensive in part because of the presence of multiple copies of ESBLs in any given clinical isolate.

Whole-genome sequencing and multilocus sequence typing has tremendous utility, as it enables microbiologists to characterize the population biology of a species, and thus track the evolution and spread of clones. Currently, these techniques are confined to reference laboratories and have little influence on local and regional infection control programmes.

### Clonality in Latin America

The CTX-M family includes a heterogeneous group of ESBLs divided into five groups based on primary structure (CTX-M-1, CTX-M-2, CTX-M-8, CTX-M-9, CTX-M-25) [26]. Although CTX-Ms are produced by a wide variety of Enterobacteriaceae strains, CTX-M globalization is associated with a few clones of *E. coli* and *K. pneumoniae*, which underscores the selective advantage of expressing these enzymes. Most clinical isolates are not clonally related, but clonal outbreaks have been described in several countries [26, 56]. It has been estimated that at least 10–20% of all UTIs are caused by clonally related *E. coli*, which are often co-resistant to aminoglycosides and trimethoprim/sulfamethoxazole [33, 56]. Detecting successful strains or epidemic clones from large volumes of isolates with the same phenotype is challenging [28].

*E. coli* belonging to phylogroup B2 [sequence type 131 (ST131)] and phylogroup D (ST405) are the most infamous community-associated, high-risk clones because of their rapid globalization coupled with high virulence and multidrug-resistant IncFII plasmids [15, 57]. *E. coli* ST131 has been implicated in severe community-acquired infections, including septicaemia [26, 58]. In Latin America, *E. coli* ST131 was initially detected in the Colombian and Brazilian hospital setting in 2008 [59, 60] and subsequently in the Colombian community setting in 2010 (along with clone ST405) and possibly in 2011 [61, 62]. The ease with which *E. coli* ST131 diffuses through communities via infected or colonized family members, wildlife, foodstuffs, and companions represents a major public health concern [15].

The composition of a different high-risk clone of *E. coli* causing community-acquired UTIs in Rio de Janeiro, Brazil, between 2005 and 2006 was determined by a cross-sectional study of 344 women seeking care in one public walk-in clinic [63]. More than half (54%) of the women had a documented UTI, of which 63% were caused by *E. coli*. Of the 50% of *E. coli* isolates resistant to ampicillin and trimethoprim/sulfamethoxazole, most (81%) belonged to 19 enterobacterial repetitive intergenic consensus (ERIC2) clonal groups. All isolates in the largest clonal group (*n* = 15 isolates) belonged to multilocus sequence typing group ST69 and phylogenetic group D, and had 89% similarity to a clonal group A (CgA) reference strain from the USA [63]. These data indicate that uropathogenic *E. coli* CgA strains have mobilized from North America and Europe to Latin America, where they have the potential to cause multidrug-resistant outbreaks.

Brazil has a particularly high diversity of CTX-M enzymes harboured by clinical isolates of *K. pneumoniae* and *E. coli*. In a community and hospital setting in Rio de Janeiro (2000–2001), analysis of the epidemiological features of 41 *E. coli* isolates resistant to third-generation cephalosporins and/or non-*β*-lactam antibiotics revealed a high prevalence of CTX-M-2 production [64]. CTX-M-9 and CTX-M-59 (a variant of CTX-M-2) were also identified. Of note, the CTX-M-producing *E. coli* in this study belonged to different phylogroups/sequence types that were associated with IncA/C plasmids implicated in the facilitation of CTX-M globalization and evolution [64].

The first report of a *Citrobacter freundii*-producing CTX-M-14 isolated from a woman in Venezuela with a recurrent UTI highlights the problem of plasmid-mediated dissemination of these *β*-lactamases into other bacterial populations [65].

## PROPOSALS FOR LATIN AMERICA

When reviewing the data, we recognize that better-designed studies are urgently required to more accurately quantify the epidemiology of Gram-negative community-associated infections in Latin America. Rather than use local criteria, these studies should report on a standardized definition for community-associated UTIs and IAIs and use approved methods for phenotypic and genotypic testing. Furthermore, local and national susceptibility data are required on a far greater number of isolates before treatment algorithms can be developed.

Even taking into consideration the gaps in our knowledge regarding the epidemiology of community-associated UTIs and IAIs by Gram-negative bacteria, the weight of evidence suggests that primary-care physicians in Latin America should consider the potential for involvement of multidrug-resistant bacteria when managing cases of UTI and IAI. Published guidelines (including recent guidelines of the Infectious Diseases Society of America for treatment of acute uncomplicated cystitis [66]) can be consulted for general guidance, but local conditions, including availability of specific agents, will influence treatment. (Fosfomycin, for example, may be used in the correct formulation for treatment of UTI in women.) Severity of infection as well as risk factors for infection by multidrug-resistant Enterobacteriaceae can be used to inform treatment decisions. If available, knowledge of local antimicrobial susceptibility data should be used as a reference guide. However, we recommend that urine cultures be collected for all recurrent or relapsing UTIs, complicated UTIs, and UTI cases presenting to the emergency room. This assertion is supported by the high levels of resistance of urinary *E. coli* isolates to trimethoprim/sulfamethoxazole and quinolones across the continent, rendering prescribing decisions difficult without supportive microbiological data [19, 39]. Urine cultures are inexpensive to perform and are readily available in most hospitals of Latin America. Furthermore, obtaining cultures is necessary to implement antimicrobial stewardship programmes, in which de-escalation is a very important method to decrease selective pressure on broad-spectrum antibiotics. Primary-care physicians, including gynaecologists, should also be educated that asymptomatic patients who receive a positive test for bacteria growth in urine should not be treated with antibiotics, as the positive test usually indicates colonization and not infection.

## CONCLUSION

Overall, data describing the microbiology of community-associated UTIs and IAIs caused by Gram-negative bacteria in Latin America over the last 10 years indicate high rates of continent-wide resistance to trimethoprim/sulfamethoxazole, quinolones, second-generation cephalosporins, and gentamicin, and low levels of resistance to third- and fourth-generation cephalosporins, nitrofurantoin, and fosfomycin. We report *E. coli* resistance rates to quinolones routinely greater than 20% (and up to 80%), which is higher than the 2009 national average in the USA (19·5%) [67]. Widespread use of quinolones in humans and in animal husbandry in Latin America may account for this difference. The concern around the endemicity of quinolone-resistant *E. coli* in Latin America is the association with plasmid-mediated quinolone resistance, which accelerates the rate at which other Enterobacteriaceae develop fluoroquinolone resistance. The extremely high rate of *E. coli* resistance to trimethoprim/sulfamethoxazole probably reflects widespread use and misuse of this low-cost antimicrobial agent for treatment of community-associated UTI in the region. Findings from the publications assessed in this review tentatively indicate that ESBL rates in *E. coli*-causing IAIs are variable but increasing over time, although not enough data are available to confirm the seriousness of this problem [43]. The rate of ESBLs harboured by urinary isolates of *E. coli* requires further study, given the ease of CTX-M mobilization, high potential for clonal outbreaks, and increase in reports of UTIs by ceftriaxone-resistant *E. coli*.

Given the high resistance rates in Enterobacteriaceae causing community-acquired UTIs and IAIs and lack of therapeutic options, we recommend that antimicrobial prescribing be guided by considering infection severity, established patient risk factors for multidrug-resistant infections, acquaintance with local antimicrobial susceptibility data, and culture collection.

## APPENDIX: Latin America Working Group on Bacterial Resistance

Carlos Alvarez (*Hospital Universitario San Ignacio and Pontificia Universidad Javeriana, Bogotá, Colombia*), Luis Bavestrello (*Clinica Reñaca, Viña Del Mar, Chile*), Eitan Berezin (*Santa Casa de São Paulo School of Medicine, Brazil*), Eduardo Gotuzzo (*Universidad Peruana Cayetano Heredia, Lima, Perú*), Manuel Guzmán-Blanco (*Hospital Privado Centro Médico de Caracas, Venezuela*), Jaime A. Labarca (*Pontificia Universidad Católica de Chile, Santiago, Chile*), Carlos M. Luna (*Hospital de Clínicas José de San Martin Hospital, Universidad de Buenos Aires, Argentina*), Carlos Mejía (*Hospital Roosevelt, Guatemala City, Guatemala*), Simone Nouer (*Hospital Universitario Clementino Fraga Filho, Rio de Janeiro, Brazil*), Eduardo Rodríguez-Noriega (*Hospital Civil de Guadalajara Fray Antonio Alcalde, Guadalajara, Mexico*), Mauro José Costa Salles (*Hospital Irmandade da Santa Casa de Misericórdia de São Paulo, Brazil*), Carlos Seas (*Universidad Peruana Cayetano Heredia, Lima, Perú*), Fortino Solórzano Santos (*Hospital de Pediatría Centro Médico Nacional Siglo XXI, Mexico City, Mexico*), Maria Virginia Villegas [*International Center for Medical Research and Training (CIDEIM), Cali, Colombia*], Jeannete Zurita (*Hospital Vozandes and Pontificia Universidad Católica del Ecuador, Quito, Ecuador*).
